# AI in Drug Discovery: Clinical Failures, Regulatory Reality, and the Validation Crisis Behind the Hype

**DOI:** 10.3390/ph19060916

**Published:** 2026-06-10

**Authors:** Lisa Khairil, Koay Hean Seng Benny, Jesreena Jerry, Farhat Mussa Khatib, Muhammad Danial Che Ramli, Suresh Kumar

**Affiliations:** 1 Department of Diagnostic and Allied Health Science, Faculty of Health and Life Sciences, Management and Science University, Shah Alam 40100, Selangor, Malaysia; ms.lisa.khairil@gmail.com (L.K.); bennykhs04@gmail.com (K.H.S.B.); jesreena26@gmail.com (J.J.); farkhatib54@gmail.com (F.M.K.); 2 Neuroscience and Mental Well-Being Centre (NeuroMIND), Management and Science University, Shah Alam 40100, Selangor, Malaysia

**Keywords:** AlphaFold, drug discovery, artificial intelligence, protein structure prediction, reproducibility crisis, clinical translation, AI bubble

## Abstract

The integration of artificial intelligence (AI) into the life sciences has accelerated significantly between 2022 and 2026, accompanied by global investment exceeding USD 100 billion and widespread expectations of a transformative impact in drug discovery. Despite these advances, the extent to which AI has improved clinical outcomes remains unclear. This study presents a structured narrative review evaluating the economic, technical, clinical, and regulatory dimensions of AI adoption in drug discovery. Current evidence indicates that clinical attrition rates remain high, with approximately 90% of drug candidates entering clinical development failing to achieve regulatory approval. Although AI systems such as AlphaFold have achieved high structural prediction accuracy, with predicted local distance difference test (pLDDT) scores exceeding 90 for well-structured proteins and root mean square deviation (RMSD) values comparable to experimental methods, limitations persist in modelling protein dynamics, post-translational modifications, and protein–ligand interactions. Clinical case studies demonstrate that while AI can accelerate early-stage discovery timelines, these advantages do not consistently translate into improved late-stage success rates. Furthermore, reproducibility challenges, limited data transparency, and regulatory gaps continue to constrain reliable implementation. These findings suggest that AI in drug discovery is currently in a transitional phase characterised by high investment but limited validated clinical impact. Future progress will depend on strengthening validation frameworks, improving data sharing practices, and aligning regulatory standards with real-world clinical performance.

## 1. Introduction

### 1.1. Evolution of Artificial Intelligence in Life Sciences

Artificial intelligence (AI) has undergone rapid development within the life sciences, progressing from traditional machine learning approaches to more advanced deep learning architectures, large-scale foundation models, and generative systems capable of designing novel biological entities. This technological evolution has been accompanied by substantial financial investment and growing expectations that AI will significantly accelerate drug discovery processes. However, the translation of computational advances into clinically effective therapies remains uncertain, as clinical attrition rates in pharmaceutical development remain high, with approximately 90% of drug candidates failing to achieve regulatory approval [1].

The evolution of AI in the life sciences can be broadly classified into four stages: (i) conventional machine learning (ML), involving statistical prediction models; (ii) deep learning (DL), employing multilayer neural networks for high-dimensional biological data; (iii) foundation models trained on large-scale multimodal datasets; and (iv) generative biology systems capable of designing novel proteins, molecular structures, and biological interactions. Machine learning methods initially enabled pattern recognition in biological datasets, while deep learning facilitated the analysis of complex, high-dimensional data such as genomic sequences and medical images. More recently, foundation models trained on large biological datasets have enabled generalisable representations, while generative models have expanded the scope of AI applications to include de novo molecular design and protein engineering [2,3].

### 1.2. Study Hypothesis and Objectives

This review hypothesises that despite substantial advances in computational capability and investment, current AI-driven drug discovery platforms remain constrained by biological complexity, limited reproducibility, and insufficient clinical validation.

## 2. Methodology

### 2.1. Literature Search Strategy

A structured narrative review methodology was employed to evaluate the clinical, technical, economic, and regulatory dimensions of artificial intelligence (AI) in drug discovery and life sciences between 2017 and 2026. Literature searches were conducted using the PubMed (National Center for Biotechnology Information, Bethesda, MD, USA), Scopus (Elsevier B.V., Amsterdam, The Netherlands), Web of Science (Clarivate Analytics, Philadelphia, PA, USA), and Google Scholar (Google LLC, Mountain View, CA, USA) databases. Additional regulatory and industry reports were retrieved from official sources including the U.S. Food and Drug Administration (FDA), European Medicines Agency (EMA), the National Institutes of Health (NIH), and major pharmaceutical or biotechnology company reports.

The search strategy combined keywords and Medical Subject Headings (MeSH) terms including: “artificial intelligence”, “machine learning”, “deep learning”, “drug discovery”, “AlphaFold”, “AI in healthcare”, “clinical validation”, “reproducibility crisis”, “AI regulation”, “FDA AI guidance”, “digital health”, “generative biology”, “protein structure prediction”. Boolean operators (“AND”, “OR”) were used to refine database searches.

### 2.2. Inclusion and Exclusion Criteria

Studies were included if they: were published between January 2017 and February 2026; were written in English; included peer-reviewed original research articles, systematic reviews, clinical trial reports, regulatory guidance documents, or high-impact industry analyses; and addressed AI applications in drug discovery, diagnostics, structural biology, clinical trials, or biomedical regulation. Studies were excluded if they: lacked methodological transparency; were non-scientific opinion pieces without supporting evidence; focused exclusively on non-biomedical AI applications; or contained duplicated or superseded findings.

### 2.3. Study Selection and Screening

Titles and abstracts identified through database searching were initially screened for relevance. Full-text articles were subsequently reviewed to determine eligibility based on the predefined inclusion and exclusion criteria. Priority was given to studies with quantitative validation, regulatory relevance, multicentre datasets, or clinically translated outcomes. In addition to peer-reviewed literature, selected regulatory guidance documents, public clinical trial databases (ClinicalTrials.gov), and financial analyses relevant to AI-biotechnology investment trends were incorporated to provide a contextual evaluation of translational and economic challenges.

### 2.4. Data Extraction and Narrative Synthesis

Relevant information relating to AI-assisted drug discovery timelines, clinical trial outcomes, regulatory developments, reproducibility concerns, validation frameworks, and economic investment trends was extracted and synthesised narratively. Emphasis was placed on identifying discrepancies between computational performance claims and clinically validated outcomes.

## 3. The Economic Dimensions of the AI Bubble

### 3.1. Investment Surge (2022–2026)

Between 2022 and 2026, the life sciences investment landscape changed substantially due to the increasing adoption of AI technologies. In 2024, global investment in healthcare reached a peak, with total funding exceeding USD 100 billion, representing an 80% increase from the previous year [4]. In the first quarter of 2025, spending on healthcare AI had nearly tripled compared to 2024 levels, reaching about USD 1.4 billion [5]. In the first half of 2025, 62% of all digital health investments went to AI-focused startups, representing a substantial increase in venture capital allocation toward AI-focused healthcare platforms [6]. Several AI-focused biotechnology firms experienced rapid valuation growth during this period, reflecting strong investor expectations regarding the potential of AI-assisted therapeutic discovery and precision medicine platforms. As part of the broader investment surge in 2026, NVIDIA and Eli Lilly announced a landmark $1 billion AI co-innovation lab aimed at transforming pharmaceutical research and development [7]. These investment patterns suggest that commercial enthusiasm for AI healthcare platforms expanded substantially faster than the accumulation of clinically validated therapeutic outcomes. Although global AI healthcare investment exceeded USD 100 billion, this still represents a relatively small proportion of the estimated USD 300–350 billion annual global pharmaceutical R&D expenditure.

### 3.2. The AI Hype Cycle in Drug Discovery

**Figure 1:**
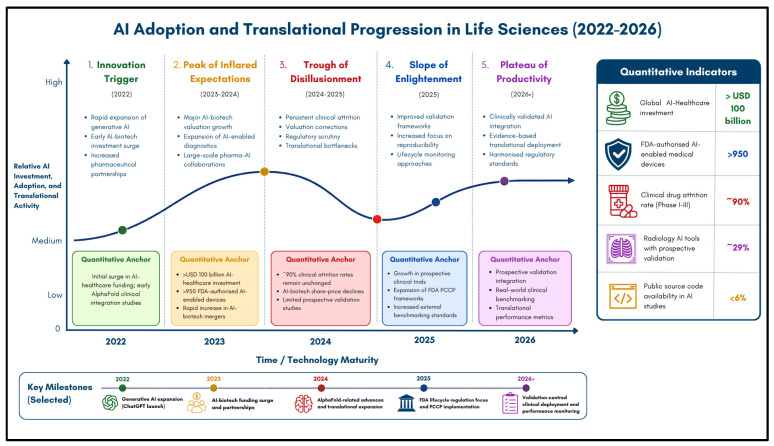
Adapted Gartner-style technology adoption curve illustrating major phases of AI expansion in life sciences between 2022 and 2026. The figure integrates representative indicators including investment growth, FDA-authorised AI-enabled medical devices, clinical attrition rates, regulatory adaptation, and translational validation challenges.

The adoption of AI in drug discovery follows a trajectory consistent with the Gartner Hype Cycle (Figure 1), characterised by an initial phase of rapid technological breakthroughs and inflated expectations, followed by a period of disillusionment driven by practical limitations in clinical translation. Early successes, particularly in protein structure prediction and molecular modelling, contributed to heightened optimism regarding the potential of AI to revolutionise pharmaceutical research. However, subsequent challenges in validation, reproducibility, and regulatory approval have tempered these expectations, highlighting a gap between theoretical capability and real-world implementation [3].

The progression through the Gartner Hype Cycle was associated with measurable indicators including venture capital inflows exceeding USD 100 billion, increasing FDA submissions for AI-enabled medical products, rapid growth in AI-biotech partnerships, and subsequent market corrections among publicly traded AI-drug discovery firms.

Examples of measurable indicators associated with these phases include venture capital investment exceeding USD 100 billion globally, more than 950 FDA-authorised AI-enabled medical devices by 2025, increasing AI-biotechnology merger activity, and persistent clinical attrition rates comparable to traditional pharmaceutical development.

### 3.3. Mega-Rounds and Market Concentration

Large funding rounds exceeding USD 100 million increasingly dominated the investment landscape during this period [8]. Large-scale funding rounds accounted for approximately 46% of total investment in digital health companies during the first quarter of 2025, indicating a strong concentration of capital among a limited number of firms. Representative examples include Xaira Therapeutics, which raised over USD 1 billion in launch funding [9], and Lila Sciences, which secured approximately USD 550 million to support AI-guided biological engineering platforms [10]. Major technology companies, including Microsoft, Google, and NVIDIA, have established strategic partnerships with biotechnology firms to capitalise on this trend [11]. As of 2023, most of the investment is still in the United States, which received USD 67 billion in AI healthcare funding, much more than China’s USD 7.8 billion [12]. There is a heavy concentration of money in a few companies and a single country. This concentration of capital may increase sector-wide vulnerability if highly valued AI-biotechnology platforms fail to achieve clinically validated translational outcomes. Overall, setbacks among a small number of dominant firms may disproportionately influence investor confidence across the broader AI-biotechnology sector.

The concentration of AI healthcare investment among a limited number of firms suggests increasing market centralisation. This trend may be quantified using the Herfindahl–Hirschman Index (HHI), commonly applied in economic competition analysis, where a small number of large funding rounds disproportionately dominated total sector investment.

Representative funding figures summarised in Table 1 were obtained from publicly reported investment disclosures and industry analyses [8,9,10,11,12].

### 3.4. Valuation Concerns and Market Corrections

Without clear evidence of a measurable return on investment, increasing signs emerged that some companies may have been overvalued as the current AI-driven valuation cycle reached its peak. The rapid growth of AI-biotech platforms between 2020 and 2026 was accompanied by unprecedented investor enthusiasm, followed by periods of valuation correction [13,14]. As once-prominent AI firms struggled to meet important clinical milestones, the market started to see many company mergers and restructuring efforts by the end of 2024. After their early clinical results failed to meet the high expectations set when they raised funding, companies like BenevolentAI, Exscientia and Recursion experienced internal changes and saw their market values decline [15]. BenevolentAI experienced substantial valuation contraction following pipeline setbacks, while several AI-biotech firms reported substantial valuation declines during 2024 market corrections. A clear example of this trend was Recursion acquiring Exscientia in 2024. Companies with strong biological research and those that were primarily driven by speculative investment enthusiasm began to be distinguished by the market after these changes. These developments suggest a shift toward investment strategies that prioritise demonstrated clinical outcomes over algorithmic novelty.

### 3.5. Return on Investment Reality

The discrepancy between substantial investment and the limited number of clinically successful AI-assisted therapeutics represents a major challenge for the current technology valuation cycle. Demonstrating a clear return on investment remains challenging despite more than a decade of substantial investment and expectations that AI would accelerate drug development. Many AI-discovered candidates fail before reaching clinical testing, and only a limited number of AI-assisted therapeutics have progressed to regulatory approval. Although AI has accelerated candidate identification and early-stage discovery workflows, it has not yet reversed the persistent increases in pharmaceutical development costs and timelines despite substantial technological advances [16]. Notably, clear evidence of improved commercial return on investment remains limited. Compared with traditional pharmaceutical development, current AI-assisted approaches have accelerated early-stage discovery but have not yet demonstrated substantially improved late-stage clinical success rates.

Recent analyses suggest that AI-assisted drug discovery platforms may reduce early-stage target identification and hit discovery timelines by approximately 30–70% compared with conventional workflows. However, despite accelerated computational screening and lead optimisation, overall clinical success rates remain broadly comparable to traditional pharmaceutical development, with estimated approval probabilities remaining within the industry baseline range of approximately 8–12%. Furthermore, available evidence indicates that reductions in late-stage development costs and regulatory timelines remain limited, as Phase II and Phase III attrition continue to be primarily driven by biological complexity, safety concerns, and insufficient clinical efficacy rather than computational inefficiency alone.

From an investment perspective, return on investment (ROI) in pharmaceutical R&D is commonly assessed using development timelines, clinical success probabilities, and overall development costs. Although AI-assisted platforms have demonstrated measurable reductions in target identification and lead optimisation timelines, evidence for proportional improvements in clinical approval rates or late-stage development efficiency remains limited. Therefore, the economic value of AI currently appears to derive primarily from early-stage productivity gains rather than from substantial reductions in overall development risk or clinical attrition.

To date, only a limited number of AI-assisted therapeutics have reached commercialisation. Therefore, direct ROI calculations based on product revenue remain premature. Thus, development timelines, attrition rates, and clinical progression metrics are currently used as surrogate indicators of economic performance.

Estimates presented in Table 2 are derived from published analyses of AI-assisted drug discovery timelines, pharmaceutical development benchmarks, clinical success rate studies, and industry assessments of pharmaceutical R&D productivity and return on investment [16,17,18,19,20,21,22,23]. Table 2 summarises currently available quantitative evidence relevant to return-on-investment (ROI) assessment in AI-assisted drug discovery.

Estimates are compiled from published analyses of AI-assisted drug discovery timelines, pharmaceutical development benchmarks, clinical success rate studies, and industry assessments of pharmaceutical R&D productivity and return on investment [16,17,18,19,20,21,22,23]. Values relating to timeline reductions, cost reductions, and projected savings should be interpreted as industry-reported or modelled estimates and may vary across therapeutic areas and development programmes.

Accordingly, these findings suggest that AI-assisted platforms may provide measurable economic benefits during the early stages of pharmaceutical R&D, including reductions in target identification timelines, lead optimisation timelines, and selected discovery-stage costs. Industry analysts have reported potential reductions in early discovery timelines of approximately 50–80%, cost reductions of 15–22%, and modelled savings ranging from USD 500 million to USD 1 billion per approved therapeutic under favourable conditions. However, despite these reported efficiency gains, current evidence indicates that overall clinical approval probabilities remain broadly comparable to conventional drug development pathways. Subsequently, the long-term return on investment of AI in drug discovery continues to depend on successful clinical translation, regulatory approval, and sustained therapeutic efficacy rather than computational efficiency alone.

A computationally promising lead compound does not necessarily translate into a clinically effective therapeutic agent. As the field matures, attention is increasingly shifting from the number of AI-designed molecules generated to their ability to demonstrate efficacy and safety in late-stage clinical development. Without consistent evidence of successful clinical translation and regulatory approval, the economic sustainability of current AI-driven drug discovery models remains uncertain. Overall, these trends suggest that although AI substantially accelerated computational workflows and investor enthusiasm, clinically validated therapeutic translation remains the principal determinant of long-term commercial sustainability. Although financial investment and commercial enthusiasm expanded rapidly, the long-term sustainability of AI in life sciences ultimately depends on whether these systems can produce measurable scientific and clinical advances. Overall, AI currently generates ROI primarily through early-stage productivity gains rather than demonstrable improvements in clinical approval rates.

## 4. Technical Achievements: Separating Signal from Noise

### 4.1. Protein Structure Prediction: The AlphaFold Phenomenon

#### 4.1.1. Genuine Breakthroughs

The emergence of AlphaFold represents a major advancement in computational biology, addressing a long-standing challenge known as the “protein folding problem” that had remained unsolved for five decades [24]. Created by Google DeepMind, AlphaFold 2 and its 2024 successor, AlphaFold 3, made substantial progress in predicting the 3D shapes of proteins based on their amino acid sequences [25]. By 2024, the AlphaFold Database had grown to include over 214 million protein structure predictions, offering a nearly complete map of the human proteome and structures from millions of other species [26]. AlphaFold-assisted structural predictions have supported target identification and structure-based screening in infectious diseases, kinase inhibitor development, and rare disease research. The developers of AlphaFold were recognised with the 2024 Nobel Prize in Chemistry for advances in protein structure prediction.

AlphaFold2 achieved median backbone RMSD values approaching experimentally derived structures in CASP14 benchmarking and routinely generated pLDDT confidence scores above 90 for structured globular proteins.

This development substantially accelerated access to structural biological information across multiple therapeutic research domains. Prior to AI-assisted structural prediction, determination of a single protein structure often required years of experimental investigation using X-ray crystallography or cryo-electron microscopy. Adding over 850 new structures, AlphaFold had a major impact on the Protein Data Bank (PDB) by 2023 [27]. Access to large-scale structural datasets has accelerated research in rare diseases, neglected tropical infections, and enzyme engineering. Overall, these advances demonstrate that AI has substantially accelerated access to structural biological information; however, the translation of these computational achievements into clinically effective therapeutics remains comparatively limited.

#### 4.1.2. Current Technical Limitations and Translational Challenges

There are still important technical challenges that are often overlooked during periods of inflated expectations surrounding AI-driven biomedical research, even though AlphaFold has been awarded a Nobel Prize. One key challenge arises from the intrinsic dynamic nature of proteins. In biological systems, proteins are highly flexible molecules that continuously undergo conformational changes essential for their function, interactions, and regulation. AlphaFold remains limited in its ability to accurately model these conformational dynamics, although this flexibility and these conformational changes are crucial for protein function [28,29]. Another limitation involves the accurate prediction of membrane protein structures [30], which are the targets for approximately half of today’s drugs [31].

Another important limitation of AlphaFold lies in its ability to accurately model the influence of metal ions, cofactors, and drug-like ligands on enzyme structure and activity [30]. Additional complications arise from post-translational modifications (PTMs), including glycosylation and phosphorylation, which can significantly alter protein structure, stability, and functional activity in vivo [32]. Accurate modelling of amino acid side-chain conformations represents another critical challenge, particularly in the context of structure-based drug design. According to studies, 7–20% of predicted side chains are frequently placed incorrectly [33]. This discrepancy highlights the continuing gap between high computational prediction accuracy and experimentally validated biological behaviour. Therefore, although AlphaFold predictions provide valuable structural insights, they may not yet fully substitute for experimentally determined structures for applications that require high-resolution accuracy in drug discovery and molecular pharmacology.

Importantly, these limitations do not diminish the major scientific contribution of AlphaFold to structural biology. Rather, they reflect the inherent complexity of modelling dynamic biological systems beyond static protein structures. Ongoing developments including AlphaFold 3, diffusion-based molecular modelling systems, and emerging platforms such as the Isomorphic Labs Drug Design Engine (IsoDDE) continue to expand AI capabilities in protein–ligand and biomolecular interaction prediction.

Released in 2024, the newest version of AlphaFold, AlphaFold 3, makes it difficult for researchers to verify the results, thereby sparking some criticism in the academic world [34] as shown in Table 3. The tension between corporate AI development and the open sharing of scientific knowledge highlights a growing concern. These findings and limitations indicate that structural prediction accuracy alone is insufficient to fully reproduce the dynamic biological complexity required for reliable therapeutic development.

Despite major advances in computational biology and structural prediction, translating these capabilities into clinically successful therapeutics has proven substantially more difficult.

### 4.2. AI in Drug Discovery: Clinical Reality Check

#### 4.2.1. The First Wave of AI-Designed Drugs

Between 2022 and 2026, several AI-assisted drug candidates entered clinical evaluation, providing the first substantive evidence of AI-driven therapeutic development [15,17]. Insilico Medicine’s Rentosertib (formerly ISM001-055; ClinicalTrials.gov identifier: NCT05938920) represented one of the earliest AI-assisted drug candidates to progress into Phase II clinical evaluation. In 2024, Rentosertib became one of the first AI-assisted drug candidates to advance into Phase IIa clinical trials for idiopathic pulmonary fibrosis [35,36]. This represented a substantially accelerated early-stage discovery timeline compared with conventional target-to-Phase I development pathways, which often require 3–6 years [18]. Despite these advances, several significant limitations and challenges remain. Multiple AI-designed drugs have faced setbacks during clinical trials [18].

Several high-profile AI drugs failed at various stages between 2023 and 2024, as shown in Table 4. Recursion Pharmaceuticals reported limited efficacy in studies involving REC-994 (ClinicalTrials.gov identifier: NCT05085561), contributing to pipeline restructuring [37]. After data from a competitor suggested limited effectiveness, Exscientia had to stop its A2A antagonist programme [38]. BenevolentAI had to reduce 30% of its workforce after the lead drug did not work, which represented another prominent attrition event [39]. DSP-1181 was another drug that was halted after its Phase I trials [40]. Approximately 90% of drug candidates entering clinical development fail to achieve regulatory approval, a rate broadly comparable to traditional pharmaceutical attrition despite AI-assisted optimisation strategies. In contrast to early expectations that AI would substantially reduce pharmaceutical attrition, current evidence suggests that biological complexity remains the dominant limitation in clinical translation. This suggests that accelerated computational optimisation alone is insufficient to overcome the biological complexity underlying late-stage clinical failure. Where available, registered clinical trial identifiers are provided to improve traceability and facilitate independent verification of AI-assisted therapeutic development programmes.

#### 4.2.2. Platform Partnership Disappointments

Long-term partnerships between AI startups and large pharmaceutical companies are a major sign that the AI investment cycle may undergo substantial correction based on their outcomes. To change how drugs are developed, substantial financial investment was directed toward these collaborations from 2012 to 2024. However, some drug targets found through these partnerships have advanced to clinical trials, with at least one entering Phase II as early as 2022 [41]. Concerns regarding limited clinical translation and slower-than-expected therapeutic progress have increasingly been raised within the industry.

Many companies that are focusing on AI are changing their approach and slowing down on big collaborations [42]. In 2024, Deep Genomics faced major doubts about its drug development plans even though it was once a leader in the field, leading to rumours about a strategy change and a potential sale of the company [43]. These partnership setbacks indicate that integrating AI into established pharmaceutical development pipelines is substantially more complex than initially anticipated. The strict evidentiary requirements for drug safety and regulatory approval continue to challenge AI-assisted development pipelines, particularly when model interpretability and biological validation remain limited. Pharmaceutical companies increasingly require clinically meaningful translational evidence rather than computational performance alone [44].

Although AI-assisted platforms have demonstrated improvements in early-stage target identification and lead optimisation timelines, downstream clinical development timelines remain largely comparable to conventional pharmaceutical development pathways. A simplified comparison between traditional and AI-assisted drug discovery workflows is summarised in Table 5.

Although AI-assisted systems have accelerated several early-stage discovery processes, these improvements have not yet consistently reduced the biological and translational barriers responsible for persistent late-stage clinical attrition.

#### 4.2.3. Fundamental Limitations

Despite substantial computational advances, AI-driven drug discovery remains constrained by limited data availability and the difficulty of accurately modelling complex biological systems. The theoretical chemical search space, estimated to exceed 10^60^ possible molecules, presents a substantial obstacle for AI-assisted drug discovery [45]. However, existing AI models have explored only an infinitesimal fraction of the vast chemical space, estimated to represent less than 10^−34^% of its theoretical diversity [46,47]. Because AI systems are inherently data-driven and trained on previously characterised compounds, they predominantly generate structural modifications of known molecular scaffolds [48]. Accordingly, these approaches tend to yield close analogues of existing drugs rather than truly novel chemotypes with the potential to address unmet medical needs or complex, treatment-resistant diseases [49].

Moreover, current computational and biophysical models remain insufficient to fully reproduce the complexity of intracellular biological systems. A concern with AI is that it is trained on data it receives, but in science, failures are often not shared. In other words, AI is trained almost exclusively on successful results, making it difficult to understand what will fail because computationally promising candidates may fail during biological and clinical validation [50]. Taken together, these limitations suggest that current AI systems remain highly dependent on the quality, diversity, and completeness of existing biological datasets.

#### 4.2.4. Implications for Medicinal Chemistry and Translational Pharmacology

The current limitations of AI-assisted drug discovery have important implications for medicinal chemistry and translational pharmacology. Although generative models can accelerate molecular design and virtual screening, optimisation of pharmacokinetic properties, toxicity profiles, metabolic stability, and off-target interactions continues to require extensive experimental validation. Many AI-generated compounds remain structurally similar to existing scaffolds, limiting true chemical novelty and reducing the likelihood of identifying first-in-class therapeutics. Furthermore, biological efficacy depends not only on molecular binding affinity but also on tissue distribution, immune interactions, pathway dynamics, and patient-specific variability, factors that remain difficult to model computationally.

### 4.3. AI in Diagnostics and Medical Imaging

#### 4.3.1. Overhyped Performance Claims

The rapid deployment of AI systems during the COVID-19 pandemic has shown a “validation gap” emerging during the current AI investment cycle in life sciences. From 2020 to 2024, thousands of AI models were quickly developed to help diagnose COVID-19 by analysing chest X-rays and CT scans. However, when experts reviewed all these models, they found a major problem: most of the hundreds of models were not clinically useful in actual medical settings because they had major challenges with how they were built. A major concern involved the use of shortcut learning, where AI systems relied on spurious correlations rather than biologically meaningful disease features [51].

Rather than identifying biologically meaningful disease features, these models often rely on spurious correlations unrelated to the underlying pathology, a phenomenon commonly referred to as shortcut learning. Researchers found that the AI was picking up clues on the patient’s position or marks on the image from the hospital setups where most patients were treated in the case of the COVID-19 model [52]. Rather than identifying pneumonia-related features, the model appeared to rely on institution-specific characteristics associated with the source of the imaging data, which is the hospital environment. The performance declined when the AI was tested with images from another hospital, showing a “generalisation gap” [53]. The decline in performance across external hospital datasets highlights persistent limitations in model generalisability and real-world clinical reliability.

The “reproducibility crisis” became a recognized problem in medical imaging by early 2025 [44]. The scientific community increasingly requires clear evidence that AI can explain its decisions and has moved from being excited about benchmark performance metrics like the “area under the curve” (AUC) metrics [54]. This is to ensure that there is a change in AI and that models focus on clinically relevant pulmonary features like ground-glass opacities, instead of picking up on non-medical things like the patient’s diaphragm or imaging noises. Computational scale alone is insufficient to ensure clinically reliable diagnostic performance, as shown in these failures. High-quality, diverse, and accurately labelled datasets remain essential for reliable model development and validation. To prevent hazardous mistakes in diagnosis, the field is now moving towards rigorous validation procedures and the involvement of human precision.

#### 4.3.2. FDA Approvals and Reality

**Figure 2:**
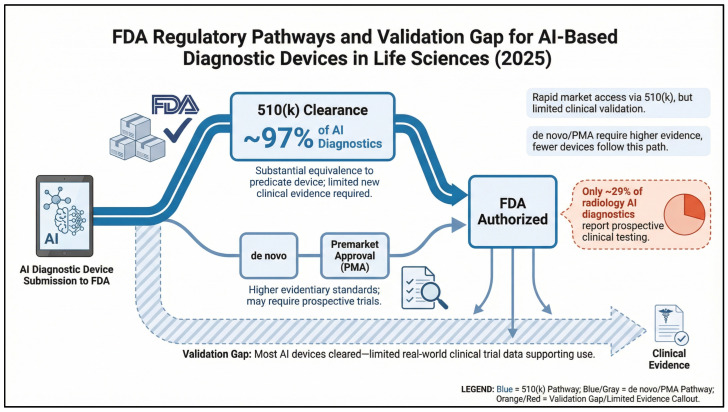
Regulatory pathways and the clinical validation gap in AI diagnostics. The schematic illustrates U.S. Food and Drug Administration pathways for AI diagnostic device authorisation. Most devices are cleared through the 510(k)-pathway based on substantial equivalence to existing devices, typically requiring limited prospective clinical evidence. In contrast, de novo and premarket approval pathways involve higher evidentiary requirements. Among radiology-specific AI diagnostics, only approximately 29% report prospective clinical testing, highlighting the gap between regulatory clearance and rigorous real-world clinical validation. These findings suggest that strong computational performance does not necessarily ensure reliable clinical generalisability.

By the end of 2024, over 210 companies were offering automated medical imaging solutions, showing that the market for AI-based diagnostic tools has grown rapidly [55]. To address concerns regarding insufficiently validated algorithms, regulatory oversight was strengthened in 2025; new rules were implemented that slowed this growth in 2025. A review by the NIH pointed to serious problems in the system even though the number of AI medical devices approved by the FDA increased numerously, reaching 950 with 76% used in radiology by late 2025 [56]. Through the 510(k) pathways, approximately 97% of AI diagnostic devices obtain FDA clearance by demonstrating substantial equivalence to previously authorised products, a process that typically requires limited new clinical evidence rather than prospective trials [57,58] as shown in Figure 2. Among radiology-specific AI diagnostics, only about 29% report undergoing prospective clinical testing [59]. This discrepancy highlights the difference between regulatory clearance and rigorous prospective clinical validation.

To address these limitations, in January 2025, the FDA released a detailed guidance titled “AI-Enabled Device Software Function: Lifecycle Management and Marketing Submission Recommendations.” A “Total Product Life Cycle” (TPLC) approach is used because this guide shifts the focus from single approvals. The performance of AI tools must be continuously monitored in real time by the companies to help spot “data drift” when the accuracy of the algorithm declines as medical practices or patients demographic shift over time [60]. For “credible” AI evidence, the guidance has set an exceptional standard, needing developers to reveal that their models work objectively for all races, genders and ages to avoid discrimination [61].

These evolving regulatory frameworks emphasise continuous lifecycle monitoring, evidence-based validation, and patient safety rather than rapid algorithm deployment alone [62,63].

## 5. Reproducibility Challenges in Biomedical AI Validation

### 5.1. Data Leakage and Overfitting Epidemic

#### 5.1.1. Data Leakage Issues

Data leakage is caused by test set information or future data points influencing model training, leading to overly optimistic performance estimates. In one article, it states that data leakage causes problems in Alzheimer’s disease research because datasets are small, imbalanced, and contain multiple measurements per subject. Samples can repeat in training and test sets, violating independent assumptions. These models may unintentionally learn subject-specific or dataset-specific patterns rather than biologically meaningful disease signatures [64,65]. Moreover, external validation, proper data splitting, and confounder control, also known as the methodological triad, are challenges faced by the current system.

Within biomedical AI validation pipelines, reproducibility challenges frequently arise from dataset heterogeneity, inconsistent preprocessing protocols, limited external validation, and insufficient reporting of model architecture and hyperparameter optimisation. These limitations may lead to substantial discrepancies between benchmark performance and real-world clinical reliability, particularly when AI systems are evaluated using small, institution-specific, or retrospectively curated datasets.

To improve reproducibility, standardised validation frameworks including external validation cohorts, prospective multicentre testing, blinded benchmarking, and adherence to reporting guidelines such as CONSORT-AI, SPIRIT-AI, and TRIPOD-AI have been increasingly recommended [66,67,68].

#### 5.1.2. Overfitted AIs

Machine learning algorithms are trained using representative datasets. However, when complex models train for too long on sample data, they integrate the irrelevant information within the dataset called ‘noise’. Noise complicates things by fitting current data too closely to the training set, causing the model to become overfitted, and it becomes unable to generalise well to new data, making it unable to perform the classification or prediction tasks for which it was intended for [69,70]. ‘Noise’ in this case is the AI learning idiosyncratic characteristics or complex patterns of the data samples that are not representative of the population.

An overfitted model can accurately represent the training data, but new data causes it to not generalise well even though it has the same distribution because some patterns in the training data are not representative of the entire population. An overfitted model is more complex than the ideal model for the data. Conversely, underfitted models perform poorly with more true generalisation error that is larger than the true generalisation error of the best possible model that can be fit with the data in hand [71].

Several strategies have been proposed to reduce the effects of overfitting. Early stopping optimises learning by stopping before ‘noise’ builds up and decreases model accuracy. Excluding noise through a “network-reduction” strategy can also allow decisions to be made faster, especially in decision tree learning models. The “data-expansion” strategy is proposed for bigger models to train their hyper-parameter sets because it requires more information to work. Lastly, the “regularisation” strategy can be used to select and keep useful features and discard less useful features that have become noise or are less impactful in the results [72].

### 5.2. Methodological Failures

Reproducibility means the results of research are always the same and consistent, no matter how many times they are repeated by independent research. The reproducibility crisis in AI is due to the AI’s inability to replicate or confirm research findings across many scientific fields [73]. Achieving reproducibility requires comprehensive documentation of training datasets, model architectures, hyperparameters, source code, and the computational environment used for model development and evaluation. These features must be systematically tracked, recorded, and standardised to ensure reproducibility [27].

The increasing model complexity of modern AI models is because each model has millions to billions of parameters with highly sensitive training dynamics and hardware-dependent behaviour. This level of complexity poses significant challenges for reproducibility, as even minor variations in model implementation, computational infrastructure, or training protocols can produce substantial differences in reported performance outcomes [74,75]. Many AI models rely on hyperparameters, such as learning rate, batch size, or regularisation strength, which need to be fine-tuned. Often, these are not shared in enough detail, or their selection is not explained rigorously, making it difficult to reproduce results. Also, slight changes in hyperparameters can result in different performance outcomes [74].

Many researchers do not publicly release source code for various reasons. It may still be under development, company owned, publication-biassed, dependent on other unpublished code, or lost. Black box means using the original code does not give reproducibility either. A recent analysis of 400 algorithms presented at two major AI conferences identified substantial limitations in research transparency and reproducibility. Only approximately 6% of studies made their underlying source code publicly available, significantly limiting independent validation and reproducibility across AI research. These findings suggest that insufficient transparency continues to limit independent validation and reproducibility across AI research. In addition, approximately one-third of the publications shared the datasets used to evaluate their algorithms, while only around half provided pseudocode outlining the proposed methods. Because pseudocode generally offers only a simplified representation of the algorithmic procedure, the absence of complete implementation details substantially limits the ability of independent researchers to reproduce, validate, and benchmark the reported findings [74]. Computational performance claims without transparent validation frameworks may overestimate the real-world biological reliability of AI systems.

### 5.3. Bias and Generalisation Problems

Bias in AI algorithms is dependent on the data they are trained on. If this data contains biases, those biases can be coded into their systems and perpetuated by the AI system. For example, if the data collected uses academic performance information that is biassed toward an ethnicity, gender, or wealth standard, the AI system could learn to pick people from that group [72]. Generalisation issues arise with idiosyncratic characteristics because some models are trained by actively selecting data instead of random sampling. Thus, the dataset is not representative of the targeted population and application goals. Poor sampling methods and poor error estimators can lead to generalisation error estimates that are biassed downward [76] and learning spurious patterns, translating into errors in the future due to a lack of information in the training data [70]. Thus, algorithms will be unable to generalise in situations that are beyond the scope of their training data.

There are also factors for biases in AI models. Data bias occurs when using unrepresentative data like gender, race, socioeconomic status, religion, or disability. Diagnostic algorithms trained on patient data in EHRs may cause unfairness in access to healthcare [77]. Since not all populations are the same, AI algorithms trained predominantly on data from specific populations may exhibit lower diagnostic accuracy for underrepresented groups, exacerbating existing health disparities [77]. Accordingly, these findings indicate that dataset imbalance may amplify existing healthcare disparities if not appropriately addressed during model development. Interaction bias is the result of improper user interactions with the model. Some doctors rely on and heavily trust an AI for a particular decision and get inaccurate advice.

The inappropriate use of an AI algorithm is known as development bias such as when patient samples are preferentially selected for the development of the algorithm [78]. In one study, 8579 digital slides from The Cancer Genome Atlas were analysed using DenseNet121 and KimiaNet for feature extraction and cancer classification. The results showed that features could identify acquisition sites with significant accuracy (70% for DenseNet121 and over 86% for KimiaNet), indicating the presence of institution-specific patterns [72].

### 5.4. Black Box Problem and Explainability

#### 5.4.1. Black Box AIs

A black box AI system refers to a non-transparent model architecture that has internal workings that become a mystery to its users. Only the system’s inputs and outputs can be read, but what happens within the AI tool to produce those outputs cannot be confirmed [79]. These AI models are trained on millions of data points through complex deep learning processes, utilising highly complex optimisation processes that are not readily interpretable by human users. How they work and produce results makes it hard to trust outputs produced by AIs [79]. Regulations such as the European Union AI Act and the California Consumer Privacy Act (CCPA), set rules on how organisations can use sensitive personal data in AI-powered decision-making tools. With black box models, it can be hard for an organisation to know whether it is compliant or to prove compliance in the event of an audit [79].

In drug discovery and molecular pharmacology, limited interpretability of deep learning systems presents substantial challenges for target prioritisation, toxicity prediction, molecular optimisation, and regulatory evaluation. Many deep neural networks can generate highly accurate predictions while providing limited mechanistic explanation regarding how specific molecular descriptors, structural features, or biological pathways contribute to the predicted outcome. This lack of interpretability complicates clinical confidence, medicinal chemistry optimisation, and regulatory assessment, particularly in applications involving adverse drug reactions, off-target interactions, and pharmacokinetic modelling. Thus, explainable AI (XAI) frameworks are increasingly being explored to improve transparency and support biologically meaningful interpretation of AI-generated predictions.

#### 5.4.2. Explainability and Interpretability

Deep learning systems used in drug discovery often rely on highly complex neural network architectures that can identify non-linear relationships between chemical structure, biological activity, and therapeutic response. However, despite strong predictive performance, these systems frequently provide limited mechanistic interpretability regarding how predictions are generated. In pharmaceutical development, this creates important challenges because regulatory approval and clinical adoption require transparent justification of molecular safety, efficacy, and biological plausibility.

**Figure 3:**
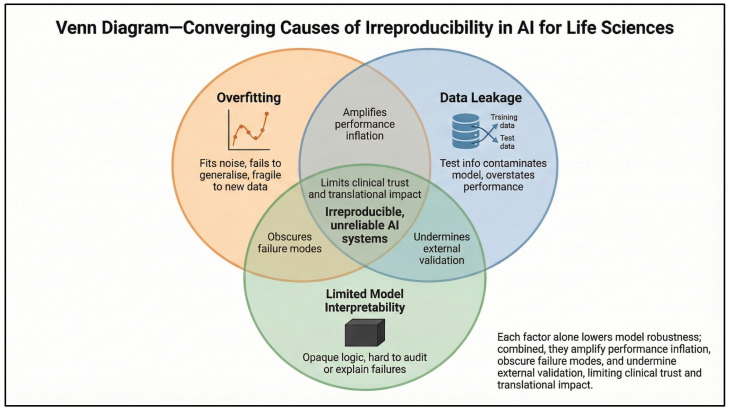
Interacting causes of the reproducibility crisis in AI for life sciences. It shows a Venn diagram illustrating how overfitting, data leakage, and limited model interpretability jointly contribute to irreproducible and unreliable AI systems. While each factor independently degrades model robustness, their interaction amplifies performance inflation, obscures failure modes, and undermines external validation, ultimately limiting clinical trust and translational impact.

Thus, explainable AI (XAI) must allow comprehensible and interpretable rules that govern a system’s decision-making process. AI systems used for target prioritisation, toxicity prediction, and clinical decision support should provide interpretable outputs. This can be independently validated by medicinal chemists, clinicians, and regulatory reviewers [80]. Within drug discovery and translational pharmacology, explainability is particularly important because clinicians, medicinal chemists, and regulatory agencies must be able to justify AI-assisted decisions involving molecular toxicity, off-target binding, metabolic stability, pharmacokinetics, and therapeutic risk. There has been an attempt to investigate various explainability methods. These include examining and describing black-box models, explaining their outcomes, and building transparent black-box models. Moreover, a taxonomy was proposed to express the underlying explanator, input data type, the issue discovered by the approach, and the “opened” black box model. It has been shown that most explanation methods are unable to decipher models [81]. Finally, establishing transparent or explicable models and fostering cross-disciplinary cooperation between healthcare practitioners are crucial for information sharing (Figure 3).

The growing recognition of reproducibility limitations and validation failures has directly influenced the development of stricter regulatory oversight frameworks.

## 6. Regulatory and Clinical Validation Challenges

Despite rapid technical advances and unprecedented investment, substantial challenges remain in getting regulatory and clinical validation. So, widespread clinical adoption of AI remains limited.

### 6.1. FDA Evolving Framework (2024–2025)

The U.S. Food and Drug Administration has been confronted with some new and unpredicted issues in regulation of medical products that integrate AI. Over 500 drug and biological products that had AI-based elements were reviewed by the agency between 2016 and 2023 [82]. This fast growth in AI-controlled submissions revealed that regulatory frameworks should be put in place which can accommodate innovation and ensure high standards of patient safety [83].

To apply AI and machine learning to drug and biological product development, in January 2025, the US Food and Drug Administration (FDA) published a draft guidance. Instead of applying equal standards of approval, they suggested a risk-based credibility assessment framework [84]. The focus of this approach is on the context of use, model transparency, data provenance and performance boundaries. The guidance presents a seven-step procedure which starts with a clear definition of the question of interest and the intended context in which the model will be used, followed by the systematic risk evaluation. It is then anticipated that developers should design and execute a plan of credibility assessment, critically record the findings and any deviations, and finally, determine whether the model is fit to be used as it is supposed to be used [85].

Nevertheless, there is less experience with AI regulation. Despite the FDA reviewing several thousands of submissions of AI-related content in medical products, a small number have gone through approval, an indication of ongoing concerns regarding generalisability, strength, and lifecycle management [86] as shown in Table 6. This pattern suggests that regulatory approval increasingly depends not only on initial algorithmic performance but also on evidence of long-term reliability, generalisability, and lifecycle monitoring. One such issue that is still unresolved is the ad hoc nature of most AI systems. The post-deployment models based on learning undermine the regulatory assumption of a locked product and raise concerns when it is necessary to re-approve or take any additional control. This concern has been one of the main obstacles to scalable clinical deployment, as pointed out by recent regulatory commentaries [87].

### 6.2. Clinical Trial Integration Issues

Although there is much laudation that AI is a method that can speed up and streamline clinical trials, its application in real clinical trial workflows has been patchy and with validation issues. Machine learning-based algorithms highly perform in a retrospective or simulated scenario but are prone to fail when implemented in a prospective manner where heterogeneity of data and protocol variations and intricacies are real and difficult to manage in clinical studies. Accordingly, regulating and trial investors have turned more sceptical regarding venturing on AI systems’ outputs without strong supporting data.

Recent studies point to the common usage of AI in clinical trials for patient screening, eligibility screening, endpoint prediction, and dose optimisation. Nevertheless, a systematic review of applications of AI in clinical trials discovered that the proportion of models that had been prospectively validated was small, with most of the studies being restricted to single-centre or retrospective data [89]. This creates generalisability issues especially when models are fitted using limited scope or historically based trial information that is not representative of contemporary clinical practice.

Also, there was a rise in regulatory demands related to the use of AI in trials [90]. Decision support systems that include AI and which could influence dosing, patient stratification, or safety surveillance have been viewed as high-risk tools that need the manifestation of explicit training information, performance boundaries, and failure modes [91]. Opinions expressed in recent regulations, as well as scholarly views, emphasise that poor validation of such systems could result in a clinically relevant mistake, particularly when there is a chance of human supervision [92,93]. All these questions have forced the authorities to underline the importance of applying human-in-the-loop (HITL) systems, particularly when it comes to interacting with AI in healthcare and escalation mechanisms in situations when AI instructions diverge with clinical perception.

Certain promising directions have been arising in the recent past, including virtual control arms and digital twins. These strategies depict the potential and the challenge of AI implementation in trials. Although the purpose of these models is to decrease the size, costs, and length of trials, they must prove biological plausibility, transparency, and reproducibility to be accepted. The methodological diversity of digital twin approaches in general and the lack of consensus on validation requirements remains, so regulatory adoption may be case-dependent [94]. In practice, most regulators are still considering AI-generated trial simulations to be supportive evidence and not a replacement for conventional clinical endpoints.

The second weakness that has remained constant is the absence of cross-institutional and multi-regional validation. Models of AI trained in an individual sponsor trial ecosystem are not usually reproducible when transferred to other sites with distinct populations of patients with varying clinical workflows or data capture behaviours. Various analyses published 2023–2025 indicate large performance losses when AI models are tested externally, which further indicates the importance of testing in multicentres before clinical/regulatory dependency [89,95]. All these issues are signs that AI cannot change the design of clinical trials profoundly. Rather, it can be said to be in the present circumstance because of its current applicability in prudently defined, strictly monitored uses, in addition to, but not as a substitution for, conventional trial methodology. Until multi-site validation becomes a possibility, trials enabled by AI are probably beneficial technology, and not determinants of regulation.

### 6.3. International Regulatory Divergence

There are significant differences in regulatory models of AI in the life sciences by jurisdiction, which impose further obstacles to cross-jurisdictional adoption. In October 2024, the European Medicines Agency (EMA) published a reflection paper in which it described a careful and well-organised position regarding AI, focusing on methodological transparency, representativeness of the dataset, and post-deployment monitoring [96]. Conversely, the UK Medicines and Healthcare products Regulatory Agency (MHRA) has developed a principles-based approach to regulation, which puts more emphasis on control over developers and allows more latitude in implementation.

This complicates multinational clinical trials and hampers international implementation of AI-enabled tools. A further aggravating factor is that proprietary AI platforms have restricted access, and do not allow independent validation and reproduction. Both academic researchers and regulators have criticised such restrictions, noting that transparency and external verification remain vital elements of clinical credibility [97].

### 6.4. Post-Market Surveillance Gaps

The challenge of ensuring the long-term safety and efficacy of AI systems is one of the major concerns even following regulatory clearance. In comparison to stationary medical devices, AI models are prone to the emergence of data drift, demographic shift, and changes in clinical practice, which may worsen performance over time [98,99]. Consequently, regulatory bodies have cautioned that AI systems that are not regulated carefully can do immense damage once they are used at scale [83,85].

**Figure 4:**
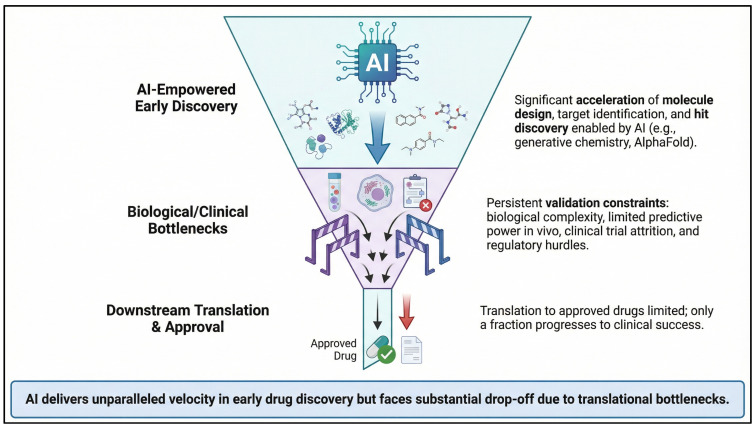
Acceleration of early drug discovery by AI and persistent translational bottlenecks. It shows a funnel illustrating the asymmetrical impact of AI across the drug development pipeline: pronounced gains in early discovery, followed by persistent biological and clinical bottlenecks that limit downstream translation and approval.

The existing post-market surveillance systems are ill-suited to the detection of these risks. The constant monitoring of performance, errors as they occur, and a formal model update process have not yet been advanced, especially when using large language models and generative systems in clinical-level settings. Recent policy reviews suggest that in the absence of well-established post-market controls, initial regulatory authorisation will only provide a partial guarantee of clinical safety and efficacy over time [100]. Such lack of approval and permanence is a serious flaw in the contemporary AI regulatory framework (Figure 4).

## 7. Critical Perspectives and Contrarian Views

### 7.1. Academic Critiques

The rapid adoption and implementation of AI in healthcare creates a situation that is different from anything in the entire history of healthcare. This form of advanced technology brings advantages and disadvantages to patients, especially when AI is installed as a dependent, semi- or fully autonomous agent in healthcare. In addition, the unclear rules for the interaction between AI and users in patient care need to be addressed in the future. Questions like the benefits, autonomy, and justice of AI and its potential to augment or interfere with the ends of medical practice must be answered to ensure AI has a place in healthcare [101]. Big data and AI initiatives also face epistemological and ontological challenges, as data generation and measurement are inherently influenced by underlying theoretical assumptions and methodological choices. Epistemological and logical problems in algorithms and issues of reliability and interpretability make them unreliable to a point. In human mental and emotional states, phenomenological problems become prevalent because the human experience is not quantifiable. These philosophical issues demonstrate several important challenges for these technologies that must be considered prior to their integration into clinical care [102].

In most studies comparing the efficiency of AI to clinicians, reproducibility issues arise. Biases are present in algorithms developed in samples coming from sources other than the ones used to train the algorithms [103]. Studies reporting AI applications in clinical practice are mostly limited to retrospective designs and sample sizes, causing selection and spectrum bias [104]. Third, few studies are known to compare AI and clinicians based on the same parameters, especially with specialty doctors [105].

Other concerns raised in this field include whether some study designs are biassed in favour of the new technology, whether the findings are generalisable, whether the study was performed in silico or in a clinical environment, and therefore to what degree the study results are applicable to the real-world setting. More than 30 AI algorithms have now been approved by the US Food and Drug Administration [105].

### 7.2. Industry Insider Warnings

One strategy for anticipating and addressing ethical challenges related to AI/ML in healthcare is patient and public involvement in the design of those technologies, also known as ‘co-design’. However, this also creates entirely new challenges. The tendency of designers to make systems more logical with an emphasis on procedures and qualities can reduce interpretability, while focusing attention primarily on the agency of patients and the public in co-design will put the design at risk. Again, there is also the risk of neglecting the broader contexts of representation and inclusion [106].

Most retrospective studies have used large numbers of patients with comparisons against expert performance by using historically labelled data to train and test algorithms. Only through prospective studies will the potential of AI systems be fully understood, because performance is likely to be worse when encountering real-world data. In addition, many studies have been published on preprint servers only and are not submitted to peer-reviewed journals, as there are few randomised controlled trials (RCTs) for AI systems. For AI systems, accuracy does not always mean applicability in clinical settings, as proven by the lack of reporting of strong numerical values [107].

### 7.3. Environmental and Ethical Concerns

In the modern era, AI is utilised in many medical fields and environments. For example, AliveCor received FDA approval in 2014 for a mobile application called Kardia, allowing smartphone-based ECG to detect atrial fibrillation [108]. Concerns regarding false positives originate from movement artefacts, and elderly patients who suffer from atrial fibrillation usually do not trust new technologies [109]. Another AI-powered tool, developed by Medtronic, received FDA approval as the Guardian system for glucose monitoring via smartphone, as diabetes becomes a growing problem [110]. The inability of some pulmonologists to interpret images compared to machine learning that was trained on pulmonary function tests is not much of a concern because proof that ML and AI can reach a final diagnosis more rapidly or more efficiently remains to be demonstrated [107]. Empatica, which developed an electrodermal captor for the detection of generalised epileptic seizures received FDA approval in 2018 for its wearable Embrace, which can alert mobile devices and trusted physicians with information about patient localisation [111]. A report focused on patient experience revealed that, in contrast to heart monitoring wearables, patients suffering from epilepsy had no barriers in the adoption of seizure detection devices and reported high interest in wearable usage [111].

A major ethical concern in AI is privacy and how data is collected, stored, and used [112,113]. This could further feed into biases, creating skewed user profiles, excluding minority groups, and providing irrelevant recommendations [114]. It is a major factor in 25.6% of the studies, where variables such as gender, racial, and age biases are all present as problems in the analysis [115]. Stereotypes, cultural insensitivity, and widening generational knowledge gaps cause AI to make biassed recommendations and outcomes [116,117]. Returning to the ‘black box’ issue, answers provided by AI are not interpretable and have no clear origin, making it hard to reproduce, while accountability of AI development is not firmly established through audit mechanisms and legal compliance. Both these factors contribute to 16.3% of the concerns [107].

### 7.4. Publication Pressure and Declining Research Quality in the AI Era

The rapid expansion of AI research in the life sciences has coincided with increasing pressure within academia to publish frequently and secure external research funding. Such pressures may inadvertently contribute to the amplification of the “AI hype cycle,” where preliminary computational findings are rapidly disseminated without sufficient validation [118]. Based on the Zurich Survey of Academics, which measures perceived pressure using six-point Likert scales, this survey examines the extent and distribution of pressure to publish and to attract external funding, and has shown that researchers in the DACH region feel a higher level of pressure to publish than to attract external funding. Pressure varies among countries and groups of academics based on factors like academic positions, culture, funds and time [119]. The quality of many publications has declined due to a variety of factors, including the emergence of paper mills. A recent analysis suggests that this ability to evade interventions is enabling the number of fraudulent publications to grow at a rate far outpacing that of legitimate science [120]. Large language model–based chatbots can generate responses with formatted citations to clinical questions. However, empirical evaluations demonstrate that these systems frequently fabricate or inaccurately attribute references, reflecting the probabilistic nature of next-token prediction rather than guaranteed factual grounding [121]. ChatGPT based on OpenAI’s GPT-3/4 large language models, can provide general references, pointing the user to textbooks and online resources, such as MedlinePlus and other websites, but it fails to provide specific details regarding the references used to generate responses [122]. Within the context of computational biology and AI-driven biomedical research, publication incentives may encourage the rapid reporting of algorithmic advances or predictive models without comprehensive experimental validation.

## 8. What Actually Works: Evidence-Based Assessment

We live in a society where using AI has become the best option to obtain data and information when one is doing research or in decision-making when it comes to the life sciences. Although AI has demonstrated benefits in life sciences research [123], significant challenges remain, particularly in clinical adoption, where integration into real-world settings is still limited [124].

### 8.1. Legitimate Applications Showing ROI

Artificial intelligence has become increasingly integrated into modern biomedical research and pharmaceutical development workflows. Life sciences generate vast amounts of complex data—genomic sequences, electronic health records, imaging scans, and clinical trial results. Traditional approaches often struggle to manage this scale efficiently. AI enables organisations to analyse, interpret, and act on this data quickly, improving decision-making across the R&D lifecycle [123].

#### Key Applications Showing Return on Investment in Life Sciences

1. Drug Discovery and Development:

AI models analyse molecular structures, predict drug interactions, and identify promising compounds faster than traditional methods, leading to a reduction in time-to-market and R&D costs while increasing the success probability. Even without compromising safety, organisations can accelerate innovation [123]. Regardless, the lack of advanced technologies limits the process of the development of drugs, making it a time-consuming and expensive task that can be addressed by using AI. AI can lead to compound and hit recognition, providing faster validation of the targeted drug and optimisation of the drug structure design [125].

2. Bioinformatics:

Bioinformatics is an important subfield in the life sciences, involving model construction and analysis of data [126]. AI in bioinformatics contributes to enhancing accuracy and data analysis efficiency [126]. Bioinformatics can be well understood as the organic integration of computer science and biology, functioning as an interdisciplinary field that encompasses research in molecular biology, mathematics, computer science, genetics and statistics [126]. Machine learning algorithms can process large-scale datasets, enabling improved predictive modelling and the identification of complex biological patterns. These developments and advancements hold immense promise for accelerating drug discovery, medicine personalisation and unlocking a deeper understanding of biological systems [127].

3. Diagnostic and Imaging:

Diagnostic medical imaging refers to a branch of medicine that visually represents different tissues and organs in the human body using various techniques for the purpose of monitoring and diagnosing health issues or conditions. The most widely used medical images are radiology, pathology, endoscopy etc. Some of these imaging techniques play an important role in the identification, detection and diagnosis of a wide range of disease such as cardiovascular, cancerous and neurological disorders by using radiological imaging techniques such as X-ray, CT, MRI, B ultrasound, positron emission tomography (PET) etc [128]. AI’s potential extends beyond surgical support and prediction of complications. Advanced AI-based algorithms enable imaging data analysis that is beyond the capabilities of human interpretation. AI assists radiologists in diagnosis and treatment planning by identifying subtle patterns and anomalies that may be difficult to detect through conventional images. The detection of subtle changes in tissue structure can be enhanced by AI-powered imaging tools, thereby aiding in the early detection of diseases and assisting in tailoring treatment plans for individual patients. AI ensures that diagnostic accuracy can evolve and become more refined over time as it can continuously learn and improve on new data [129].

### 8.2. Incremental Improvements vs. Revolutionary Claims

The influence of AI on the life sciences in 2025 is known and defined by a tension between incremental operational advantages and revolutionary claims in discovery. The overall industry transformation from experimental workflows to enterprise-wide impact is still ongoing. Although AI technologies have contributed to reducing the duration of certain early-stage processes, particularly in target identification and molecular screening, the broader transformation from isolated computational tools to enterprise-wide integration across the biomedical research and pharmaceutical development pipeline is still evolving. Accordingly, the current landscape reflects a hybrid model in which AI augments traditional experimental approaches rather than fully replacing them. Recognising this distinction is essential for maintaining realistic expectations regarding the role of AI in drug discovery, clinical translation, and the broader life sciences ecosystem.

#### 8.2.1. Revolutionary Claims

The application of AI in the life sciences provides insight into how computational technologies may transform healthcare delivery and biomedical research. Actively, it works to accelerate research, personalise medicine and improve patient care. Even though proponents argue that AI is shifting the life sciences fundamentally from a trial-and-error approach to a predictive computational-first paradigm [130]. AI has reduced optimisation phases from years to months and shortened target discovery timelines [131]. For example, in less than 18 months, Insilico medicine developed a lead fibrosis candidate compared to standard multi-year methods [132]. Clinical success data show that AI-designed compounds have phase 1 from 2024 to 2025 had a success rate of 80% to 90% [133]. Scientific innovation, such as the creation of new proteins, e.g., esmGFP, demonstrated that AI has enabled breakthroughs such as simulating 500 million years of molecular evolution [134].

#### 8.2.2. Incremental Improvements

Sceptics and pragmatists point out that AI remains a tool for efficiency rather than a total paradigm shift for much of the industry [18,124]. Despite quicker discovery, the regulated clinical trial procedure continues to be a significant obstacle. With an emphasis on AI for mundane tasks such as patient recruitment, medical writing and record keeping, AI’s influence in this context is sometimes characterised as incremental. Clinical challenges are not always associated with and correlated with discoveries. In May 2025, Recursion Pharmaceuticals stopped using its top AI-discovered candidate (REC-994) since long-term data did not support previous efficacy trends. For sophisticated AI analytics, a significant portion of healthcare data remains fragmented and of low quality, limiting success [135,136]. Many organisations struggle to convert their AI strategies into measurable outcomes, even though 93% of businesses intend to boost their AI budgets by 2025 [137,138].

### 8.3. Successful Integration Models

AI provides some valuable insights that inform strategic decision-making and drive innovation. Due to their sheer volume and velocity, data collected from sensors and Internet of Things (IoT) products are complex and vast. These data streams encompass diverse types of information, ranging from environmental conditions and equipment status to user interactions and preferences, which require precise data analysis to enable better decision-making [139].

AI, as a non-human intelligence programmed to complete specific tasks, can overcome some of the computationally intensive and intellectual limitations of humans. For example, AI could be a computer application that is competent to solve a complicated business problem for managers. AI-enabled systems generate personalised recommendations through the analysis of large- enhances scale clinical and biomedical datasets. Thus, it is believed that AI could be smarter than the best humans and experts in any field. The value of using AI tools is perceived based on the trade-off between possible benefit and risk; when the benefit is higher than the risk, a greater value of using the technology is perceived [139,140].

In terms of drug discovery within the “Closed-Loop” R&D Model, AI significantly efficiency in drug discovery by analysing vast chemical and biological datasets to predict pharmacological properties, including activity, toxicity, and adverse effects, thereby accelerating compound screening and optimisation. For instance, AI-driven drug discovery platforms identify potential anticancer agents within significantly reduced timeframes. Mirroring artificial neural network (ANN) architectures (computing systems inspired by biological neural networks) used for learning assessment in AI-enhanced educational settings, multilayer neural networks similarly optimise candidate molecule selection in pharmaceutical screening. Generative AI models such as Insilico Medicine’s Chemistry42 platform, integrated with reinforcement learning, designed the novel idiopathic pulmonary fibrosis inhibitor INS018_055, taking 18 months from target discovery to preclinical candidate nomination [132]. As INS018_055 represents a paradigm shifting advance in AI-driven drug discovery, exemplified by its efficient identification of the TNIK kinase target, its trajectory spanned from discovery to Phase II trials within a four-year timeframe, supported by rigorous multimodel therapeutic validation [124].

The optimisation of patient pathways reflects a shift towards outcome-based simulation models. The management of clinical trials is evolving through AI. The usage of synthetic Control Arms (SCAs) is one of the major developments. Researchers can now simulate control groups instead of recruiting large numbers of people for placebo groups using historical data and real-world evidence (RWD). This addresses many of the ethical issues associated with placebo testing, and not only reduces the need for human subjects [141]. Also, AI is tackling the challenge of recruitment of patients. Electronic health records (EHRs) can be scanned by tools such as TrialGPT to find the right candidates instantly, potentially increasing enrolment by up to 25% potentially and reducing the time it takes to fill a study. Finally, through adaptive study design, trials are becoming more flexible [142]. By analysing data in real time, researchers can refine patient populations in the middle of the study or modify dosages [143].

## 9. Lessons from Previous Hype Cycles

### 9.1. Historical Parallels

Computational biology was the earliest use of AI in the biological sciences [144], as a result of breakthroughs, the area has grown in recent years due to learning algorithms and the greater availability of data [145]. Currently, the life sciences business employs a wide range of AI models, each having unique strengths and complexity.

The foundations for AI were established by fundamental concepts such as the Turing Test and the first artificial neural networks in the 1950s [146,147]. The 1960s and 1970s witnessed the creation of one of the earliest artificial neural networks, the Adaptive Linear Neuron (ADALINE), as well as a better understanding of single-layer neural network limitations. The 1980s saw significant developments in deep learning, including early types of cognitive computing, model-free reinforcement learning, and backpropagation in neural networks [148,149,150]. By the 1990s, the first practical applications of AI in drug development had emerged [151,152].

With important turning points like the creation of convolutional neural networks (CNNs) and recurrent neural networks (RNNs), the 2000s saw the emergence of deep learning models. Natural language processing underwent a revolution in the 2010s and 2020s with the advent of transformer-based models and generative adversarial networks (GANs). For example, IBM created a question-answering system in 2007 called Watson, which was able to outcompete top contestants and champions on the television show Jeopardy. This system used DeepQA, which used language processing to analyse data from different contexts and extract information from a wide array of sources to arrive at an answer. This created an opportunity for applications in the healthcare field, as inputs no longer needed to be limited to symptoms and outputs could be more complex than pure clinical diagnosis. For example, in 2017, the Watson system was able to determine RNA-binding proteins that were associated with amyotrophic lateral sclerosis. New systems were made to support patient care in various capacities. Pharmbot, for example, was developed in 2015 to provide education regarding medication and treatment processes for patients and their families [153].

AI applications in the life sciences were modest, with computational models assisting in basic data analysis and genetic sequencing. The emergence of bioinformatics was witnessed in the late 20th century, where AI algorithms played a part in the analysis of vast amounts of biological data and in their deciphering [145,154]. The discovery and diagnosis of diseases began to take shape as genomics and proteomics accelerated. In terms of the identification of potential drug candidates, machine learning algorithms proved instrumental in analysing complex genetic patterns and predicting biological interactions [155].

### 9.2. The Productivity Paradox

The productivity paradox refers to the observation that productivity has not increased proportionally despite the increased investment in technology and AI. This can be due to different causes, such as the time required for personnel to adjust to new technologies. Importantly, the productivity paradox shows that real-world results may fall behind expectations while AI can potentially increase productivity [156]. By 2025, the AI productivity paradox in the life sciences is characterised by a growing divide between technological capability and clinical output, where regulatory, organisational, and operational barriers prevent overall development timelines from significantly improving despite faster individual research processes [157].

The delivery of new medicines remains largely unchanged, even though drug discovery has accelerated substantially. AI advances have enabled the identification of drug candidates in as little as 46 days, reducing early-stage discovery timelines from several years to roughly one year [158]. However, this speed has not extended downstream. The overall path from discovery to market approval still averages more than 10 years, as preclinical validation, regulatory evaluation and clinical trials continue to progress at traditional rates [159]. This speed gap is compounded by a pronounced investment mismatch. In the pharmaceutical sector, AI investment continues to be heavily directed toward drug discovery, with comparatively less focus on operational domains such as scale-up, manufacturing, and supply chain management, even though AI has clear potential to impact these areas through quality control and distribution optimisation [160,161]. Consequently, rapid computational discovery has yet to translate into faster patient access, revealing a structural disconnect between real-world discovery and technological progress.

A new set of operational bottlenecks has become increasingly apparent by 2025, where a ‘triple tax’ has emerged as a major supervisory burden. Outputs generated by AI systems, such as clinical trial reports or medical documentation, frequently contain subtle errors or hallucinations. As a result, the time saved during content generation by professionals is offset by the intensive human effort required to correct, verify and assume legal responsibility for the final output [157]. Legacy integration has emerged as another significant constraint at the organisational level. Established life sciences companies report greater short-term productivity losses than younger AI-native biotech’s; productivity can initially reduce by approximately 1.33% as integrating AI tools with legacy laboratory equipment and fragmented data silos introduces technical complexity [162]. The problem in clinical settings is further compounded by alert fatigue and the proliferation of false tasks. AI-driven early warning systems, such as those used for sepsis detection, have been reported to generate false alert rates as high as 95% [163].

In clinical settings, the problem is further compounded by alert fatigue and the proliferation of false tasks. AI-driven early warning systems, such as those used for sepsis detection, have been reported to generate false alert rates as high as 95% [163]. These high false-positive rates have triggered unnecessary laboratory tests, diverting staff time and clinical evaluations and resources without corresponding improvements in patient outcomes [163,164].

### 9.3. Market Correction Mechanisms

The most significant constraint on AI-driven discovery is no longer computational capacity, as suggested by a growing body of evidence, but the pace at which predictions can be experimentally verified. Contemporary AI systems generate hypotheses at a rate that far exceeds the laboratory’s ability, whether robotic or human, to test them. Platforms such as AlphaFold and generative chemistry models in drug discovery have produced vast numbers of plausible structures and targets. Yet, within realistic timeframes, only a small fraction can be validated empirically. This imbalance has resulted in what some researchers describe as “hypothesis overflow,” whereby hypotheses are generated at a rate that exceeds the capacity for experimental and chemical validation [129,165].

This bottleneck has increasingly been framed by recent research as a limitation of existing validation infrastructure, rather than a failure of AI. In response, a parallel shift has begun to take shape, centred on automation in experimental verification. In studies published between 2024 and 2025, advances were highlighted in robotic laboratories, shared platforms designed to support independent replication across institutions, and closed-loop experimentation [166]. These systems aim to address concerns that untested AI generator results may exacerbate existing reproducibility problems in the life sciences and to reduce the lag between confirmation and prediction.

Market-based governance mechanisms alongside these technical corrections are playing a growing role in disciplining AI development. To manage risk, economic signals are being used rather than relying exclusively on investors, insurers, formulation regulation and scientific institutions. The emergence of AI-specific audits and insurance frameworks is one of the manifestations of this trend, in which developers are required to maintain documentation, monitor performance before coverage is offered, demonstrate validation processes and documentation. Analysis from academics and policymakers suggests that these mechanisms translate technical uncertainty into financial costs and foster transparency even in the absence of prescriptive rules [167].

A widening gap between clinical returns and demonstrable financial returns was noted from recent reviews, prompting investors to differentiate more carefully between speculative promise and validated value [168]. Due diligence processes have become more exacting, particularly in the life sciences as enthusiasm surrounding AI has matured. Investor behaviour has evolved; claims of AI maturity are increasingly scrutinised against evidence of regulatory readiness, reproducibility and real-world performance rather than proof-of-concept demonstrations alone.

This recalibration has been further supported by efforts to reduce information asymmetry. Standardised disclosure tools such as datasheets for datasets and model cards are now widely discussed as practical mechanisms for improving transparency between AI developers and end-users in clinical settings and in scientific fields. More informed adoption and critical evaluation, built upon frameworks covering training data characteristics, intended use, limitations, and known failure modes are increasingly viewed as foundational to responsible AI deployment [169].

At a macroeconomic level, these corrective pressures are also visible. Central banks and financial authorities have cautioned that AI-related valuations may face adjustment as expectations realign with demonstrable productivity gains. Periods of rapid technological investment are often followed by repricing once infrastructure costs and realised returns diverge, as warned by economic analyses from central banking institutions. Large technology firms have begun to reassess the scale and timing of investment in AI-specific infrastructure in parallel, signalling a shift from expansion driven by anticipation to one guided more closely by return on investment.

In financial systems dominated by automation, comparable corrective dynamics have long been recognised. In the United States, algorithmic trading in equity markets is now responsible for most of the transaction volume. It has also been subjected to increasingly stringent oversight following episodes of extreme volatility, including flash crashes. Tightly coupled algorithmic responses to shared signals can amplify instability, as demonstrated by empirical analyses, reinforcing the need for human oversight, circuit breakers and structural constraints on automated systems [170]. These lessons have increasingly been invoked in discussions of AI governance beyond finance, underscoring the broader risks of unchecked automation operating at machine speed.

These developments suggest that the current phase of AI adoption is characterised less by unchecked acceleration than by a gradual process of correction. Institutional safeguards, technical bottlenecks and economic discipline are converging to temper early optimism and ensure sustainable impact, shifting the emphasis from the volume of output to verifiable clinical outcomes and reproducible scientific evidence.

## 10. Structural Barriers to AI Success in Life Sciences

### 10.1. Biological Complexity vs. AI Capabilities

Although AI has significant potential in the life sciences, its practical applicability remains constrained by the complexity of biological systems. Biological processes are inherently non-linear, dynamic, and context-dependent, operating across molecular, cellular, tissue, and organismal levels [171]. The resulting complexity creates feedback loops and emergent behaviours that AI models, especially deep learning systems, are unable to capture [172,173]. Although it is possible to draw correlations in training datasets using these models, the models often fail to provide a cause-and-effect relationship or extrapolate to new biological setups, including patient-specific drug reactions or multi-omics interactions [172,173]. In drug discovery, these models have the capability to predict molecular binding affinity but cannot explain the broader bioactivity in vivo, hence demonstrating the disconnect between prediction and the biological reality of computational drug discovery [174,175,176]. Also, the vast size of chemical and biological space further complicates the model training process, exposing it to overfitting, and restricting its validity in new conditions [177].

### 10.2. Data Quality and Availability

Another important obstacle is data constraints. AI needs big and good-quality datasets for training, but biological and clinical data are often partial, unavailable and skewed. These data come in many different forms, such as clinical records, high-throughput sequencing, and imaging, which have different formats and standards. This heterogeneity causes inconsistency in performance and model reproducibility. Ownership of data silos and regulation limits access to the various datasets required for model training. Datasets may not be standardised in their data formats and curation protocols, making it difficult to achieve inter-integrated and cross-institutional validation. Furthermore, these problems are aggravated by the curse of dimensionality in high-throughput and multi-omics data because many variables are frequently larger than the number of samples, compromising predictive accuracy [174,175,177]. The response to these barriers involves data standardisation, curation and collaborative sharing initiatives.

### 10.3. Validation Infrastructure Deficits

Experimental validation infrastructure also helps to limit the predictive power of AI. Predictions that are done in the computer should be confirmed by wet-lab experiments, but some institutions do not have the capability to run prospective confirmations. Wet-lab testing is still time-consuming, expensive and technical, especially in the case of translating predictions to in vitro, in vivo or clinical environments. A lack of standardisation of validation pipelines and frameworks of reproducibility also reduces the trust in AI-generated insights. Indeed, even sophisticated models do not yield consistent results because of inconsistencies in preprocessing of data, model training, or experimental conditions, which further reminds us of the need for robust and scalable validation strategies [175,177]. The lack of such infrastructure would mean that AI models will be theoretical resources instead of tools in actionable use in biological research.

### 10.4. Organisational and Cultural Factors

Lastly, organisational and cultural issues also play a key role in the adoption of AI in life sciences. The lack of alignment between developers of technologies and biologists usually results in either overpromising in publications or fund requests, which sets inaccurate expectations of AI functions. Not all teams are interdisciplinary; computational scientists are unaware of biological subtleties, and life scientists do not know programming languages or deal well with model outputs, which might result in further misunderstanding of these model outputs and a decrease in the integrative nature within the research workflow. Also, a culture in which negative outcomes are not valued prevents critical insights into AI constraints, hindering the acceleration of iterative improvement. These barriers can only be overcome through cross-disciplinary training, congruent incentives where emphasis is focused on reproducibility and validation and a culture of openness in reporting positive and negative results [174,178,179].

## 11. The Path Forward: Realistic Expectations and Best Practices

### 11.1. Recalibrating Expectations

The solution to the problem of a sustainable future for AI in the life sciences is to purposefully readjust expectations. However, instead of making AI appear as an alternative to experimental science or clinical experience, there is growing evidence of how AI can serve as an augmentative aid that supports human decision-making processes and speeds up certain aspects of the research process. Unless AI is applied to create sustainable value in the life sciences, then expectations should be recalibrated to represent biological and translational reality, and not technological optimism. The literature is also experiencing a steady subscription to AI-guided drug discovery tests, indicating that many claims of success are a posteriori and clubbish in nature with minimal prospective or trial validation yet [174].

With AI being more integrated into research in the life sciences, one of the priorities should be to legitimise expectations to the point that they are more aligned with biological realities than the hype of technology. Surveys of AI applications in the life sciences regularly categorises an extensive number of existing applications as comprehensively effective on retrospective datasets yet show less evidence of an obvious effect on real-world biological results, such that statements about a near-term breakthrough transformation should be accepted cautiously [180]. Although applications like protein structure prediction platforms have revolutionised certain fields (e.g., AlphaFold has allowed structural analysis on millions of proteins), it is still humans who need to interpret such applications and have experimental data to convert them into biological insights or therapeutic discoveries. The future of AI is more sustainable when it is placed as an augmentation tool that improves hypothesis generation, gives more priority to experimental work, and provides a faster way to carry out research as opposed to an alternative method to experimental science.

This recalibration also highlights the inability of the context-dependent functions of biological complexity, emergent behaviours and multi-scale interactions to be fully explained using only static or black-box models. In realistic scenarios, the focus is on slow but steady improvements to existing technology and the imperative to ensure effective validation, instead of visualising AI as a solution to all problems in life sciences [124].

### 11.2. Recommended Research Priorities

Research priorities should switch their focus to robustness, transparency, and cross-domain applicability to get substantive contributions instead of hype. There is an increasing agreement in the field that explainable AI (XAI) methods are necessary so that models can be made interpretable and that model predictions are significant to domain experts when they involve safety, efficacy, or biological processes. Toxicology reviews and computational prediction landscapes point to the importance of explainability coupled with causal inference and multi-modal learning to be more useful with complex biological data [181]. Also, life sciences data are often heterogeneous, noisy, and biassed. Additionally, structural inconsistencies such as structured and sporadic missingness across datasets hinder robust model training and generalisation, because integration across sources remains technically challenging [182]. Before focusing on improving transferable and reproducible AI models, prioritising the curation of structured data, harmonisation and joint annotation norms will help. Notably, forward-looking validation systems with models being evaluated using new data prior to implementation must become the norm. Open validation pipelines, interlaboratory validation, and systematic comparisons to experimental validation can enhance confidence in AI predictions and overcome the disparity between computational and biological performance [183].

### 11.3. Policy and Regulatory Recommendations

Policy and regulatory ecosystems must adapt to support responsible and credible AI integration in life sciences. Regulatory bodies increasingly acknowledge that AI systems, particularly those embedded in drug development or clinical decision support, require lifecycle-based oversight that includes risk assessment, validation documentation, performance monitoring, and quality management throughout model deployment [184]. For instance, perspectives in regulatory science emphasise transparency, explainability, and interpretability as essential for regulatory credibility and trustworthiness, echoing calls for Good Machine Learning Practice in regulated environments [185].

Transparency and explainability are not merely regulatory requirements but fundamental components of stakeholder trust. Regulators, healthcare professionals, and patients require insights into how AI systems generate decisions, particularly in high-stakes contexts such as drug approval, personalised treatment recommendations, and clinical risk prediction. Several international initiatives, including consensus guidelines for trustworthy AI in healthcare, have outlined core principles such as fairness, traceability, usability, robustness, and explainability, which may help inform governance frameworks aligned with ethical, clinical, and legal standards [186]. Moreover, harmonising regulatory approaches across different jurisdictions will help streamline AI validation and reduce duplication of effort, enabling more efficient adoption of AI tools that meet robust standards for safety, efficacy, and equity.

### 11.4. Investment Strategy Shifts

Lastly, income distribution in AI in the life sciences needs to change. The previous investment waves were fuelled by hype and speculative hopes as opposed to solid biological or clinical achievements. Recent market surveys and strategic focuses show that there is an emerging trend towards evidence-based and milestone funding, where less emphasis is placed on disruptive proof points, reproducibility, and direct contributions to research processes (such as improved prediction of drug interactions or integration with regulatory science, instead of overall assertions of revolutionary effect) [187].

Firms are also becoming aware that a longer investment horizon that coincides with the nature of drug development, regulatory approval, and clinical trials turns out to be more realistic and would yield long-term returns. The balance of risk through the diversification of funding portfolios into several focused streams of research is also useful, as it is well known that high attrition rates are a typical feature of the life sciences and that often promising AI models do not pass preclinical trials but continue to be informative for future innovations.

## 12. Conclusions: Beyond the Bubble

AI has rapidly transformed multiple areas of the life sciences, particularly protein structure prediction, diagnostics, and early-stage drug discovery. Despite substantial advances in computational capability and unprecedented investment, whether these technologies can consistently improve clinically meaningful outcomes remains uncertain. This review demonstrates that although AI systems have substantially accelerated target identification, molecular screening, and structural prediction, these improvements have not yet translated into proportional reductions in clinical attrition rates or regulatory complexity. Persistent challenges involving reproducibility, limited external validation, biological complexity, model interpretability, and insufficient data transparency continue to constrain reliable clinical implementation. The major disconnect between computational acceleration and biological validation represents the defining limitation of current AI-driven drug discovery. Although AI can efficiently generate hypotheses and optimise molecular candidates, downstream therapeutic success remains dependent on highly complex biological systems that are not yet fully captured by existing computational models. By integrating technical, economic, clinical, regulatory, and reproducibility perspectives, this review provides a multidisciplinary assessment of the validation crisis underlying contemporary AI enthusiasm in the life sciences. Rather than rejecting AI-driven innovation, the findings highlight the importance of shifting emphasis from computational novelty alone toward clinically validated translational performance. Future progress will depend on rigorous prospective validation, transparent benchmarking practices, representative datasets, reproducible methodologies, and internationally harmonised regulatory frameworks. Sustainable integration of AI into drug discovery will ultimately require balancing technological innovation with evidence-based clinical translation.

## Figures and Tables

**Table 1:** Major AI-focused biotechnology and drug discovery companies involved in recent high-value investment and partnership activity.

Company	Year	Verified Funding/Deal Size	Funding Stage/Type	Core AI Platform	Strategic Significance
Xaira Therapeutics	2024	Over USD 1 billion committed capital	Launch funding & venture backing	Foundation-model-driven drug discovery	One of the largest AI-biotech launches to date, integrating machine learning, biological data generation, and therapeutic development into a unified AI-native pharmaceutical platform.
Lila Sciences	2025	USD 550 million total funding (USD 350 M Series A; USD 200 M prior funding)	Series A + strategic AI investment	Autonomous AI Science Factories	Develops “scientific superintelligence” using AI-guided robotic laboratories capable of autonomous experimentation across biotechnology, materials science, and chemistry.
Isomorphic Labs	2024–2026	USD 2.1 billion Series B (2026) + earlier strategic pharma collaborations worth ~USD 3 billion potential milestones	Series B + pharmaceutical partnerships	AlphaFold-derived AI drug design engine (IsoDDE)	DeepMind spin-off applying structural AI and predictive molecular modelling for drug discovery; advancing AI-designed therapeutics toward human clinical trials by late 2026.
Pathos AI	2025	USD 365 million	Series D	Multimodal oncology foundation models	Integrates clinical, molecular, imaging, and pathology datasets to optimise precision oncology and improve clinical trial patient stratification.
Recursion Pharmaceuticals	2024–2026	USD 150 million + strategic AI and pharma expansion	Public biotech + strategic collaborations	Phenomics-driven AI drug discovery	Combines automated wet-lab experimentation with large-scale phenomics and machine learning pipelines to accelerate therapeutic discovery and validation.
Insilico Medicine	2024–2026	Multi-billion-dollar pharmaceutical collaboration potential including Eli Lilly agreements	Strategic pharmaceutical partnerships	Generative AI for target and molecule design	Recognised for advancing AI-generated drug candidates into clinical development and reducing preclinical discovery timelines using generative chemistry platforms.
Generate Biomedicines	2024–2026	USD 700 million + cumulative funding and strategic collaborations	Venture funding + pharma partnerships	Generative protein foundation models	Applies machine learning foundation models to design novel therapeutic proteins and biologics with programmable functional properties.
Exscientia	2024–2026	Multi-billion-dollar collaboration ecosystem	Public biotech + pharma alliances	AI-guided precision medicine platform	Among the earliest AI-native drug discovery firms to advance AI-designed molecules into human clinical studies, validating AI-assisted medicinal chemistry workflows.
Absci	2024–2026	USD 300 million + cumulative funding and partnerships	Public biotech + strategic collaborations	Generative AI biologics design	Integrates synthetic biology, protein engineering, and AI foundation models for rapid antibody and biologic therapeutic development.
Genesis Therapeutics	2024–2026	USD 280 million + cumulative funding	Series B and strategic investment rounds	Geometric deep learning for molecular discovery	Uses physics-informed and geometric AI models to improve structure-based small-molecule drug design and target prediction.
Eikon Therapeutics	2024–2026	Over USD 1 billion cumulative funding	Venture capital + strategic expansion	AI-enhanced live-cell imaging analytics	Combines super-resolution live-cell imaging with machine learning to analyse protein dynamics and accelerate therapeutic discovery pipelines.
Charm Therapeutics	2025–2026	USD 70 million + Series A funding	Series A	DragonFold generative molecular AI	Develops 3D graph neural network models for structure-aware drug discovery, especially in oncology and difficult-to-drug targets.

**Table 2:** Quantitative ROI-relevant metrics comparing conventional and AI-assisted drug discovery platforms.

ROI-Relevant Metric	Conventional Drug Discovery	AI-Assisted Drug Discovery	Economic/ROI Implication
Target Identification and Validation	Often requires several years of experimental screening and validation	AI can prioritise targets within months by integrating multi-omics, the literature, and biological network data	Faster target identification reduces early R&D expenditure and opportunity costs
Lead Generation/Hit Discovery	Typically, 2–4 years from target to optimised lead candidate	AI-assisted platforms have reported reduction to months–1 year in some programmes	Accelerates project progression and reduces labour-intensive screening costs
Compound Screening Capacity	Limited by laboratory throughput and cost	Millions to billions of virtual compounds can be screened computationally before wet-lab testing	Reduces experimental burden and increases productivity per researcher
Cost per Approved Drug	Frequently estimated at USD 1–2.6 billion including failures	AI aims to reduce attrition-related costs throughout discovery and development	Lower failure rates substantially improve investment efficiency
Potential Savings per Successful Drug Programme	Baseline cost structure maintained	Modelling studies estimate >USD 1 billion potential savings per approved drug if AI reduces failure rates across stages	Represents one of the largest projected ROI benefits of AI adoption
Lead Optimisation Time	Traditional medicinal chemistry cycles may require years of iterative optimisation	Industrial implementations report approximately 50% reduction in lead optimisation time	Earlier entry into clinical development improves net present value (NPV)
Phase I Clinical Success Rate	Historical industry average ~40–65%	AI-discovered molecules reported ~80–90% success in Phase I	Improved early clinical success reduces capital lost through attrition
Phase II Clinical Success Rate	Approximately 30–40% historically	AI-discovered molecules reported ~40% in current analyses	Suggests potential but not yet definitive improvement in later-stage ROI
Overall R&D Productivity	High attrition remains a major cost driver	Improved candidate selection may increase overall probability of success and portfolio productivity	More assets can reach clinical development with similar resources
Current Evidence for Realised ROI	Established market data available	Limited because few AI-discovered drugs have reached commercial approval	Most ROI evidence remains projected or early-stage rather than fully realised

Current evidence suggests that AI provides the greatest economic benefits through accelerated target identification, lead optimisation, virtual screening, and reduced attrition rates. However, definitive long-term ROI remains under evaluation because relatively few AI-discovered drugs have completed the full development and commercialization cycle. Comprehensive ROI assessments will require larger numbers of AI-discovered drugs to complete regulatory approval and commercialization.

**Table 3:** Technical Comparison of AlphaFold 2 and AlphaFold 3.

Feature	AlphaFold 2 (AF2)	AlphaFold 3 (AF3)	Critical Limitations/Implications
Primary Release	2021	2024	AF3 represents a paradigm shift beyond protein-only structure prediction
Prediction Scope	Single proteins and protein complexes	Proteins, protein–protein, protein–DNA/RNA, protein–ligand complexes	AF2 limited to polypeptides; AF3 expands biochemical context but still not full cellular realism
Underlying Architecture	Evoformer + Structure Module (MSA and pairwise attention)	Unified diffusion-based generative model	AF3 improves flexibility but increases computational complexity
Multiple Sequence Alignment (MSA) Dependence	Strong dependence	Reduced dependence	AF2 struggles with orphan proteins and low-homology targets
Membrane Proteins	Partial success; often inaccurate loop orientation and transmembrane packing	Improved modelling with explicit environment-aware representations	Neither version fully accounts for lipid bilayer dynamics or membrane heterogeneity
Post-Translational Modifications (PTMs)	Not supported	Limited implicit handling (e.g., ligands, cofactors)	Critical limitation: phosphorylation, glycosylation, acetylation, ubiquitination is not explicitly modelled
Intrinsic Disorder Regions (IDRs)	Poorly resolved; low confidence scores	Slightly improved flexibility modelling	Still inadequate for highly dynamic or phase-separating proteins
Protein–Ligand Interactions	Not supported	Explicit ligand and small-molecule modelling	Binding affinities, kinetics, and induced fit remain unreliable
Conformational Dynamics	Single dominant conformation	Ensemble-like generative outputs	Neither captures time-dependent conformational switching
Complex Stoichiometry	Fixed, user-defined	More flexible complex assembly	Cannot predict biologically correct stoichiometry de novo
Environmental Context	No cellular context	Partial biochemical context	Lacks pH, ionic strength, crowding, and redox environment modelling
Accuracy (Globular Proteins)	Very high (near experimental)	Comparable or improved	Accuracy drops sharply for flexible, multi-domain systems
Clinical/Drug Discovery Utility	Target structure prediction	Target–ligand hypothesis generation	Still insufficient alone for lead optimisation without experimental validation

**Table 4:** Clinical performance of first-wave AI drugs. Data compiled from company disclosures, ClinicalTrials.gov records, investor reports, and published analyses [15,17,40].

Drug Candidate	Company/Platform	Indication	Clinical Phase	Outcome/Status	Notes
Rentosertib (ISM001-055)	Insilico Medicine	Idiopathic pulmonary fibrosis (IPF)	Phase IIa → Phase IIb/III planning	Positive efficacy signal; advancing	AI-discovered TNIK inhibitor showed dose-dependent FVC improvement (~trend up to ~100 mL) with acceptable safety. First widely recognised AI-designed molecule reaching meaningful Phase II signal; larger confirmatory trials ongoing.
DSP-1181	Exscientia/Sumitomo Pharma	Obsessive–compulsive disorder (OCD)	Phase I	Discontinued after Phase I	First AI-designed drug tested in humans; completed Phase I safety evaluation but failed to demonstrate sufficient clinical progression → terminated after Phase I.
DSP-0038	Exscientia	Psychosis/neuropsychiatric disorders	Phase I	Ongoing/completed early Phase I	CNS serotonin receptor modulator; early clinical safety evaluation completed/ongoing depending on cohort; no efficacy data yet.
EXS-21546	Exscientia/(post-merger Recursion ecosystem)	Solid tumours (A2A receptor antagonist)	Phase I	Early clinical stage	First-in-class AI-designed immuno-oncology agent targeting adenosine signalling; Phase I safety/PK evaluation ongoing; no efficacy readouts yet.
EXS4318	Bristol Myers Squibb/Exscientia	Autoimmune/inflammatory disease	Phase I	Early clinical development	Licenced PKC-θ inhibitor; Phase I initiated with early safety/PK signals only; no efficacy results reported.
REC-994	Recursion Pharmaceuticals	Cerebral cavernous malformation	Phase II	Discontinued (lack of efficacy)	Phase II safety acceptable but failed to show meaningful clinical efficacy → programme terminated during pipeline prioritisation (2025).
REC-2282/REC-3964	Recursion Pharmaceuticals	NF2/*C. difficile* infection	Phase II/preclinical	Deprioritised/discontinued	Multiple early assets removed during portfolio optimisation; reflects challenge in translating phenomics AI hypotheses into clinical efficacy.
REC-1245	Recursion Pharmaceuticals	Solid tumours/lymphoma (RBM39 degrader)	Phase I/II	Active early clinical evaluation (2026)	Dose escalation ongoing; 2026 updates show good safety, predictable PK, no DLTs, early signals still pending efficacy validation.
REC-4881	Recursion Pharmaceuticals	Familial adenomatous polyposis (FAP)	Phase II	Emerging strong efficacy signal (2026)	2026 updates show clinically meaningful polyp reduction signals and regulatory engagement for potential registrational pathway.
Additional AI pipeline expansion (Recursion–Exscientia merged ecosystem)	Recursion/Exscientia integrated platform	Oncology, immunology, CNS	Phase I–II mixed	Expanding portfolio (2025–2026)	Post-merger platform now includes multiple Phase I–II programmes; shift toward AI-native “OS-driven drug discovery” pipeline scaling.

**Table 5:** Comparison of traditional and AI-assisted drug discovery development timelines and translational outcomes.

Development Stage	Traditional Drug Discovery	AI-Assisted Drug Discovery	Current Limitation
Target identification	~2–4 years (target hypothesis → validation)	Months to ~1 year (computational + omics + ML prioritisation)	Experimental validation still required; AI improves prioritisation but does not eliminate false targets
Hit discovery	~1–2 years	Weeks to months using generative models + virtual screening	Binding prediction accuracy still imperfect for complex protein dynamics
Lead optimisation	~1–3 years	~30–70% time reduction reported in AI-assisted pipelines	Limited generalisation across chemotypes; ADMET prediction uncertainty remains
Preclinical development	~1–2 years	Moderate acceleration (in silico toxicity + PK filtering)	In vivo toxicology and regulatory studies remain mandatory
Clinical trials (Phase I–III)	~6–8 years	No consistent time reduction observed yet (2026 evidence)	Regulatory constraints + human biology dominate; AI impact minimal in late-stage duration
Overall development timeline	~10–15 years	~8–12 years (no systematic reduction yet, but faster entry to clinic)	AI mainly improves earlier pipeline speed, not approval speed
Clinical success rate (overall approval probability)	~8–12% (industry average)	Similar (~8–12% overall, based on current AI pipelines)	AI has not yet reduced Phase II/III attrition significantly
Key real-world evidence	Traditional pipelines dominate approvals	DSP-1181 (failure), REC-994 (failure), Rentosertib (Phase II signal), REC-4881 (emerging signal)	AI improves discovery but clinical translation bottleneck persists

**Table 6:** FDA AI/ML regulatory framework for AI-enabled medical products, including Total Product Life Cycle (TPLC) monitoring, risk-based credibility assessment, and post-market surveillance requirements according to FDA requirements for AI trustworthiness [88].

Regulatory Component	Step/Requirement	Description	Regulatory Intent/Implication
Risk-Based Credibility Assessment	Step 1: Define the Context of Use (COU)	Clearly specify how the AI model is used (decision support, automation, diagnosis, triage) and its role in clinical decision-making	Anchors the level of regulatory scrutiny to clinical risk
	Step 2: Identify Model-Informed Decision (MID)	Determine what clinical or regulatory decisions rely on the AI output	Ensures traceability between model output and patient impact
	Step 3: Assess Risk Level	Classify potential patient harm if the model fails (low, moderate, high risk)	Drives proportional validation and evidence requirements
	Step 4: Establish Credibility Goals	Define acceptable performance, uncertainty bounds, and reliability thresholds	Prevents “black box” deployment without performance guarantees
	Step 5: Verification (Technical Validation)	Confirm the model is correctly implemented and computationally sound	Addresses software errors, data leakage, and reproducibility
	Step 6: Validation (Clinical Relevance)	Demonstrate the model accurately reflects real-world clinical behaviour using appropriate datasets	Central FDA requirement for AI trustworthiness
	Step 7: Applicability and Uncertainty Analysis	Evaluate generalisability, bias, and robustness across populations and settings	Mitigates risks of demographic bias and dataset shift
Total Product Life Cycle (TPLC) Monitoring	Pre-Market Performance Evidence	Submission of training data characteristics, model architecture, and validation results	Establishes baseline safety and effectiveness
	Algorithm Change Protocol (ACP)	Predefined plan describing allowable model updates and re-training strategies	Enables controlled post-market learning systems
	Post-Market Performance Monitoring	Continuous monitoring for performance drift, bias, and unexpected behaviour	Recognises AI as a dynamic, non-static medical product
	Real-World Data (RWD) Integration	Use of clinical deployment data to reassess safety and effectiveness	Aligns AI regulation with learning healthcare systems
	Transparency and Documentation	Model versioning, audit trails, and explainability documentation	Supports regulatory audits and clinical accountability
	Human Oversight Requirements	Defined clinician-in-the-loop or human-on-the-loop controls	Prevents over-automation in high-risk clinical contexts
	Corrective and Preventive Actions (CAPA)	Mandatory response plans for detected failures or adverse events	Ensures rapid mitigation of patient safety risks

## Data Availability

No new data were created or analysed in this study. Data sharing is not applicable.

## Abbreviations

The following abbreviations are used in this manuscript:

ACP	Algorithm Change Protocol
AF2	AlphaFold 2
AF3	AlphaFold 3
AI	Artificial Intelligence
AUC	Area Under the Curve
CAPA	Corrective and Preventive Actions
CCPA	California Consumer Privacy Act
COU	Context of Use
CT	Computed Tomography
EHR	Electronic Health Record
FDA	U.S. Food and Drug Administration
FVC	Forced Vital Capacity
IDR	Intrinsically Disordered Region
IND	Investigational New Drug
IPF	Idiopathic Pulmonary Fibrosis
LLM	Large Language Model
MID	Model-Informed Decision
MSA	Multiple Sequence Alignment
NIH	National Institutes of Health
PDB	Protein Data Bank
PCCP	Predetermined Change Control Plan
PTM/PTMs	Post-Translational Modification(s)
RWD	Real-World Data
TPLC	Total Product Life Cycle
ToS	Terms of Service
VC	Venture Capital
XAI	Explainable Artificial Intelligence
